# Endoscopic treatment of walled-off necrosis with fistulas to the small intestine and colon using a lumen-apposing metal stent

**DOI:** 10.1055/a-2794-7734

**Published:** 2026-03-24

**Authors:** Shin Yagi, Sho Hasegawa, Yu Honda, Takeshi Iizuka, Yusuke Kurita, Kensuke Kubota, Masato Yoneda

**Affiliations:** 1 218758 Department of Gastroenterology and Hepatology, Yokohama City University Hospital, Yokohama, Japan

Walled-off necrosis (WON) is a recognized complication after acute pancreatitis 1 , and the usefulness of endoscopic treatment with lumen-apposing metal stent (LAMS) placement using interventional endoscopic ultrasound (IV-EUS) has been reported 2 . WON has also been reported to occasionally form fistulas with the adjacent gastrointestinal tract 3 . We report the first case of WON with fistulas to both the small intestine and the colon successfully treated by LAMS placement using IV-EUS ( Video 1 ).

Endoscopic treatment of walled-off necrosis with fistulous communication to both the small intestine and the colon, successfully performed by lumen-apposing metal stent placement using interventional endoscopic ultrasound.Video 1

**Fig. 1:**
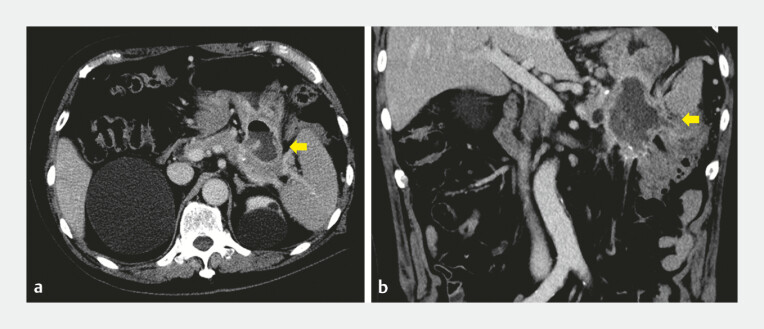
Computed tomographic images showing walled-off necrosis adjacent to the stomach. Axial ( **a** ) and coronal ( **b** ) images.

**Fig. 2:**
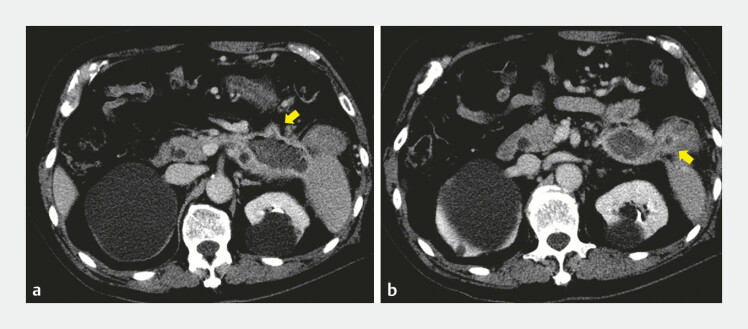
Computed tomographic images demonstrating fistulous communication between the walled-off necrosis and the small intestine ( **a** ), and between the walled-off necrosis and the colon ( **b** ).

**Fig. 3:**
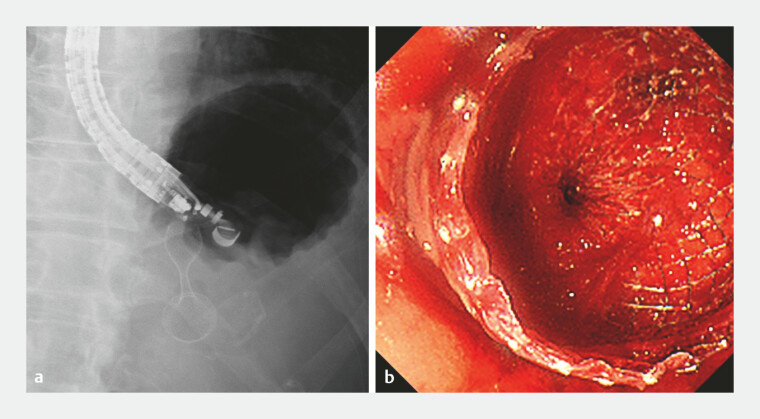
A radiographic image showing lumen-apposing metal stent placement ( **a** ) and the corresponding endoscopic image after stent deployment ( **b** ).

**Fig. 4:**
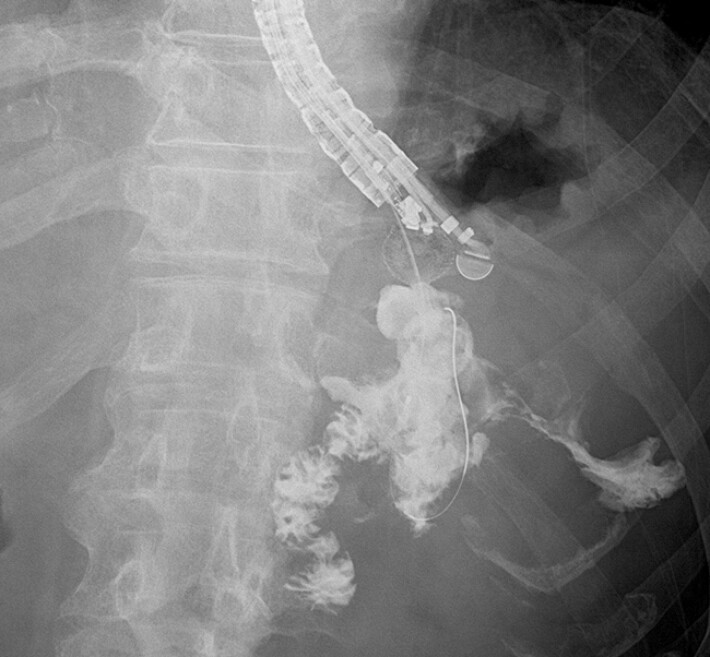
A radiographic image showing contrast injection into the walled-off necrosis through the lumen-apposing metal stent, demonstrating communication with both the small intestine and the colon.

**Fig. 5:**
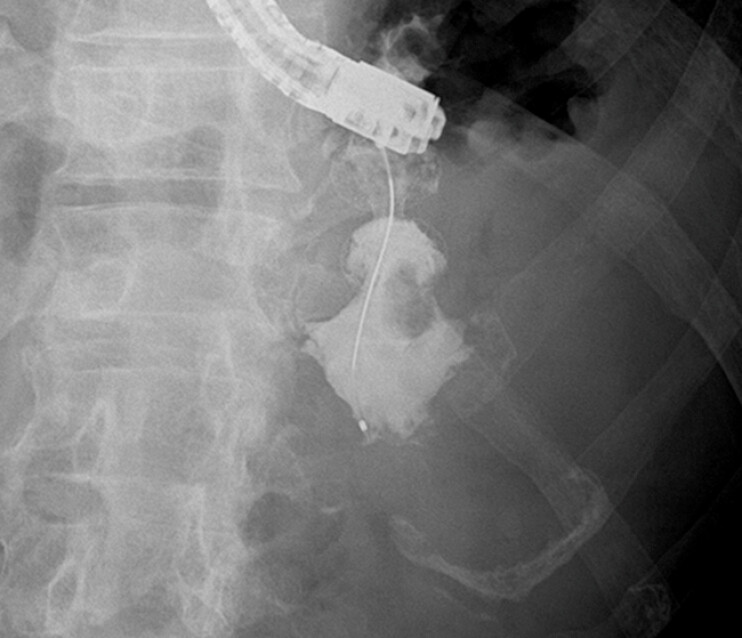
A radiographic image showing reduction of the walled-off necrosis and resolution of fistulas to the small intestine and the colon after lumen-apposing metal stent placement.

An 82-year-old man developed alcohol-induced acute pancreatitis and subsequently formed WON. He was admitted with abdominal pain and elevated inflammatory markers, suggesting infected WON. Computed tomography demonstrated WON adjacent to the stomach ( Fig. 1 ), with suspected fistulous communication to both the small intestine and the colon ( Fig. 2 ). After discussion with surgeons, endoscopic treatment was selected as the initial therapeutic approach. A LAMS (Hot AXIOS, 15 mm, 1 cm; Boston Scientific, Marlborough, MA, USA) was placed transgastrically into the WON using IV-EUS. After stent deployment, a catheter was inserted into the WON through the LAMS ( Fig. 3 ), and contrast injection demonstrated opacification of both the small intestine and the colon via the fistulas ( Fig. 4 ). After LAMS placement, the patient’s abdominal symptoms and inflammatory markers improved, and no procedure-related adverse events were observed. Six weeks after LAMS placement, the WON had decreased in size, and a decision was made to remove the LAMS. A catheter was reinserted through the LAMS and contrast injection was performed, which demonstrated no contrast leakage and no fistulous communication with either the small intestine or the colon ( Fig. 5 ). The LAMS was subsequently removed. During a 6-month follow-up period after stent removal, no recurrence of WON or fistula formation with the gastrointestinal tract was observed.

This is the first reported case of WON with fistulas to both the small intestine and the colon successfully treated by LAMS placement using IV-EUS, avoiding surgical intervention.

Endoscopy_UCTN_Code_TTT_1AS_2AJ
